# Bidirectional associations between chronic diseases and Long COVID: a population-based cohort and interrupted time-series analysis

**DOI:** 10.3389/fpubh.2026.1834448

**Published:** 2026-07-16

**Authors:** Hanna Sõnajalg, Martti Vanker, Kristi Läll, Märt Möls, Maris Alver, Lili Milani, Kai Kisand, Pärt Peterson, On behalf of the Estonian Biobank Research Team

**Affiliations:** 1 Estonian Genome Centre, Institute of Genomics, University of Tartu, Tartu, Estonia; 2 Institute of Mathematics and Statistics, University of Tartu, Tartu, Estonia; 3 Institute of Biomedicine and Translational Medicine, University of Tartu, Tartu, Estonia

**Keywords:** asthma, COPD, Long COVID, polygenic risk score (PRS), type 2 diabetes

## Abstract

**Background:**

Long COVID has been associated with an increased incidence of chronic diseases, including type 2 diabetes (T2D), which has been suggested to have a bidirectional relationship with COVID-19. We examined these associations in a population-based cohort.

**Methods:**

Chronic non-communicable diseases (NCDs) diagnosed before (2010–2019) or after (2021–2023) the COVID-19 pandemic onset were tested for associations with Long COVID among Estonian Biobank participants (*N* = 2,639 with Long COVID, *N* = 199,171 controls) using logistic regression. Interrupted time-series analysis using a SARIMA model assessed changes in monthly T2D incidence after March 2020. A polygenic risk score (PRS) for severe COVID-19 was examined for association with new-onset T2D.

**Results:**

Pre-existing NCDs were associated with Long COVID, and individuals with Long COVID had increased odds of first-recorded NCD diagnoses after 2020. Long COVID was associated with COPD (OR = 2.33), asthma (OR = 2.18), and chronic kidney disease (OR = 2.15). It was also associated with higher odds of incident asthma (OR = 5.12), COPD (OR = 4.08), and sleep apnea (OR = 3.05), while the association with T2D did not surpass Bonferroni correction. However, T2D incidence increased after March 2020 beyond expected trends (*p* = 1.84 × 10^−80^). Individuals in the highest PRS quantile for severe COVID-19 had increased odds of T2D (OR = 1.19).

**Conclusion:**

The findings support bidirectional associations between COVID-19 and NCDs, including T2D. Pre-existing T2D was associated with increased odds of Long COVID, while the pandemic period showed increasing trends in T2D incidence.

## Introduction

Recent studies estimate that a substantial proportion of individuals, ranging from 10-30% of non-hospitalized and 50–70% of hospitalized cases, develop post-acute sequelae of SARS-CoV-2 infection, commonly referred to as Long COVID, after recovering from the initial illness (1–3). Among the various complications associated with Long COVID, cardiovascular diseases (4), pre-existing chronic respiratory diseases such as asthma and chronic obstructive pulmonary disease (COPD) (5) and chronic kidney disease (CKD) have been associated with a higher risk of Long COVID (6). Among these conditions, metabolic diseases, and particularly type 2 diabetes (T2D), have received increasing attention due to their bidirectional relationship with COVID-19 outcomes.

Pre-existing T2D is consistently associated with worse outcomes after SARS-CoV-2 infection, including increased hospitalization, intensive care unit admission, and mortality (7–12). Individuals with T2D also appear to have a higher risk of developing Long COVID; a UK cohort study reported a greater likelihood of persistent Long COVID symptoms among people with T2D compared to those without diabetes (13). Interestingly, SARS-CoV-2 infection has also been linked to a higher probability of T2D remission in individuals recently diagnosed with the disease (14). Beyond its impact on pre-existing diabetes, COVID-19 has been associated with increased incidence of new-onset T2D. In a cohort of 629,000 individuals tested for SARS-CoV-2, diabetes incidence showed a 3–5% excess burden over a median follow-up of 257 days (15). Meta-analyses report a significantly elevated diabetes risk (RR = 1.62–1.64) following COVID-19 compared to non-infected controls (7, 16, 17). Similarly, a Korean population-based study found a 30% higher risk of new-onset diabetes after COVID-19 (18), and a large English cohort of 16 million individuals demonstrated increased T2D incidence among those hospitalized with COVID-19 (19). These findings are consistent with a relationship between COVID-19 and T2D, however, most studies focus on short-term outcomes, and data on the long-term burden of T2D and its complications after COVID-19 remain limited.

In this study, we investigated the role of chronic diseases, including T2D, as risk factors for Long COVID using the Estonian Biobank (EstBB), comprising 210,000 individuals with linked health records and genotyping data. We further analyzed whether the incidence of chronic diseases associated with severe SARS-CoV-2 infection, such as T2D, increased during the pandemic period (2020–2023).

## Methods

### Study participants

The study was performed using data from EstBB, a volunteer-based biobank of the Estonian adult population (≥18 years old), with a current cohort size of more than 210,000 individuals and representing the Estonian adult population in terms of age, sex, and geographic distribution. EstBB is regularly linked to national registries, hospital databases, and the national Health Insurance Fund, which provide comprehensive information on reimbursed healthcare services at the national level. Further details about the EstBB cohort and genotyping procedures have been published elsewhere (20). The study focused on individuals with the Long COVID condition or post-viral fatigue syndrome (ICD-10 codes U09/U09.9 or G93.3, respectively), defined between June 2020 and December 2023. The ICD-10 code G93.3 was included because it was frequently used to code chronic fatigue-like symptoms that many COVID-19 patients experienced during long-term recovery. The controls were EstBB individuals without a Long COVID diagnosis, however, the majority were likely exposed to SARS-CoV-2 during the pandemic period between March 2020 and 2023. These individuals were analyzed for 12 non-communicable diseases (NCD) represented by 28 ICD-10 codes documented in EstBB (Supplementary Tables S1, S2).

### Statistical analysis

#### Association between pre-existing and new-onset NCDs and Long COVID

Logistic regression was used to test associations involving the 12 NCD groups separately, as well as any of the diseases combined, adjusting for birth year and sex as covariates. For analyses of pre-existing disease, NCD diagnoses recorded between 2010 and 2019 were used as exposures and Long COVID diagnosis during 2020–2023 as the outcome. For analyses of new-onset disease, Long COVID diagnosis was used as the exposure and first-recorded NCD diagnoses during 2021–2023 as outcomes, restricting the analysis to individuals without the respective diagnosis before 2020. Sensitivity analyses were conducted that additionally included BMI as a covariate. The BMI measurement closest prior to March 2020 was used. Because multiple disease groups were evaluated, statistical significance was assessed using a Bonferroni-corrected threshold of *α* = 0.0042 (0.05/12).

#### Interrupted time series analysis

We conducted an interrupted time series analysis following Kurvits et al. (21) to examine the impact of the COVID-19 pandemic on monthly incidence rates of newly diagnosed T2D. Using a seasonal autoregressive integrated moving average (SARIMA) model with external covariates, we assessed longitudinal changes while accounting for autocorrelation and seasonal variation (22, 23). March 2020 marked the start of the pandemic period, coinciding with Estonia’s national emergency declaration. We analyzed data from January 2012 to February 2020 (pre-pandemic) and from March 2020 to December 2023 (pandemic). To model the pandemic’s effect, we included three intervention terms: (i) a pulse indicator for the sharp drop in cases during March–May 2020, (ii) a step term for a sustained shift in incidence, and (iii) a ramp function for gradual trend changes (23). Seasonality was modeled using calendar month, and a linear time trend accounted for long-term changes (Supplement Figures S1A,B). The optimal SARIMA model was selected using the auto.arima function (R package forecast [39]), prioritizing the lowest Akaike Information Criterion (AIC) and minimal residual variance. Model fit was confirmed with residual diagnostics and the Ljung–Box test. We model the interrupted time series using regression model: with autocorrelated errors. Specifically,


$$Z_{t}=\beta_{0}+\sum_{m=2}^{12}\delta_{m}D_{m,t}+\beta_{1}t+\beta_{2}{\text{pulse}}_{t}+\beta_{3}{\text{ramp}}_{t}+\beta_{4}{\text{step}}_{t}+Y_{t}$$

Where $Z_{t}$ denotes the number of incident T2D diagnoses at time t , $D_{m,t}$ are monthly dummy variables capturing deterministic seasonal effects and t represents time in months. The intervention variables represent a temporary pulse effect (pulse), a gradual post-intervention trend change (ramp) and a permanent level change (step) associated with the COVID-19 pandemic. The regression errors $Y_{t}$ follow a seasonal ARMA process, specifically a SARMA $(1,0,2)\times {\left(1,0,1\right)}_{12}$ model:


$$(1-\phi_{1}B)(1-\Phi_{1}B^{12})Y_{t}=(1+\theta_{1}B+\theta_{2}B^{2})(1+\Theta_{1}B^{12})\varepsilon_{t}$$

Where B denotes the lag (backshift) operator, defined by $B^{k}Z_{t}=Z_{t-k}$ , and { $\varepsilon_{t}$ } is a white-noise process with mean zero, constant variance $\sigma^{2}$ and no serial correlation. To obtain the counterfactual trajectory in the absence of the COVID-19 pandemic, a separate regression model: excluding the COVID-related intervention variables was fitted using data up to February 2020. This baseline model includes monthly dummy variables and a linear time trend and is combined with a seasonal ARMA structure SARMA $(1,0,1)\times {\left(1,0,1\right)}_{12}$ . To estimate the cumulative T2D burden attributable to the pandemic, we summed up the predicted contributions of the intervention terms and calculated a 95% confidence interval using standard errors, assuming parameter independence. Analyses were conducted in R 4.4.2 (RStudio), with significance set at a two-sided *p*-value < 0.05.

#### Association with the polygenic risk score for severe COVID-19

Genetic predisposition to severe COVID-19 was quantified using a polygenic risk score (PRS) derived from GWAS summary statistics for the B2 phenotype (hospitalized COVID-19 vs. population controls) from the COVID-19 Host Genetics Initiative (24). Summary statistics from the meta-analysis, excluding the 23andMe and EstBB cohorts to avoid sample overlap, were used. All EstBB participants were genotyped using Illumina GSAv1.0 and GSAv2.0 arrays, as well as GSAv2.0_EST and GSAv3.0_EST arrays with add-on SNV content from EstBB whole-genome sequencing data, aligned to the GRCh37 reference genome. Quality control followed best practices. Pre-phasing was carried out with Eagle v2.3 and imputation with Beagle v.28Sep18.79367 (25) using the Estonian population-specific reference panel based on 2,695 whole-genome sequencing samples (26). The COVID-19 PRS was calculated using a Bayesian polygenic prediction approach (PGS-CS) that models linkage disequilibrium and applies continuous shrinkage to single-variant effect sizes. Analyses were restricted to well-imputed autosomal single-nucleotide variants included among 1.1 million HapMap variants (27) and an external European sample-based LD reference from the 1,000 Genomes Project (28). The resulting PRS was standardized to a mean of 0 and standard deviation of 1, and individuals of European ancestry were subsequently stratified into low (0–20%), intermediate (20–80%), and high (80–100%) PRS quantiles for downstream analyses. Logistic regression was used for testing for an association between incident T2D and COVID-19 PGS, adjusting for birth year, sex, and 4 first genotype principal components. A sensitivity analysis was conducted that additionally adjusted for BMI.

## Results

### Characteristics of the study population

The study included 201,810 Estonian Biobank participants with available genotype and electronic health records, of whom 2,639 had a recorded Long COVID diagnosis and 199,171 served as controls. Individuals with Long COVID were predominantly female (75.3%) and had a mean age of 49.5 years and mean BMI of 27.5. Controls were 65% female, with a mean age of 46.4 years and mean BMI of 26.5. Long COVID cases were most common among individuals aged 40–59 years (42.7%) (Supplementary Tables S1, S2).

### Association between pre-existing NCDs and Long COVID

**Figure 1:**
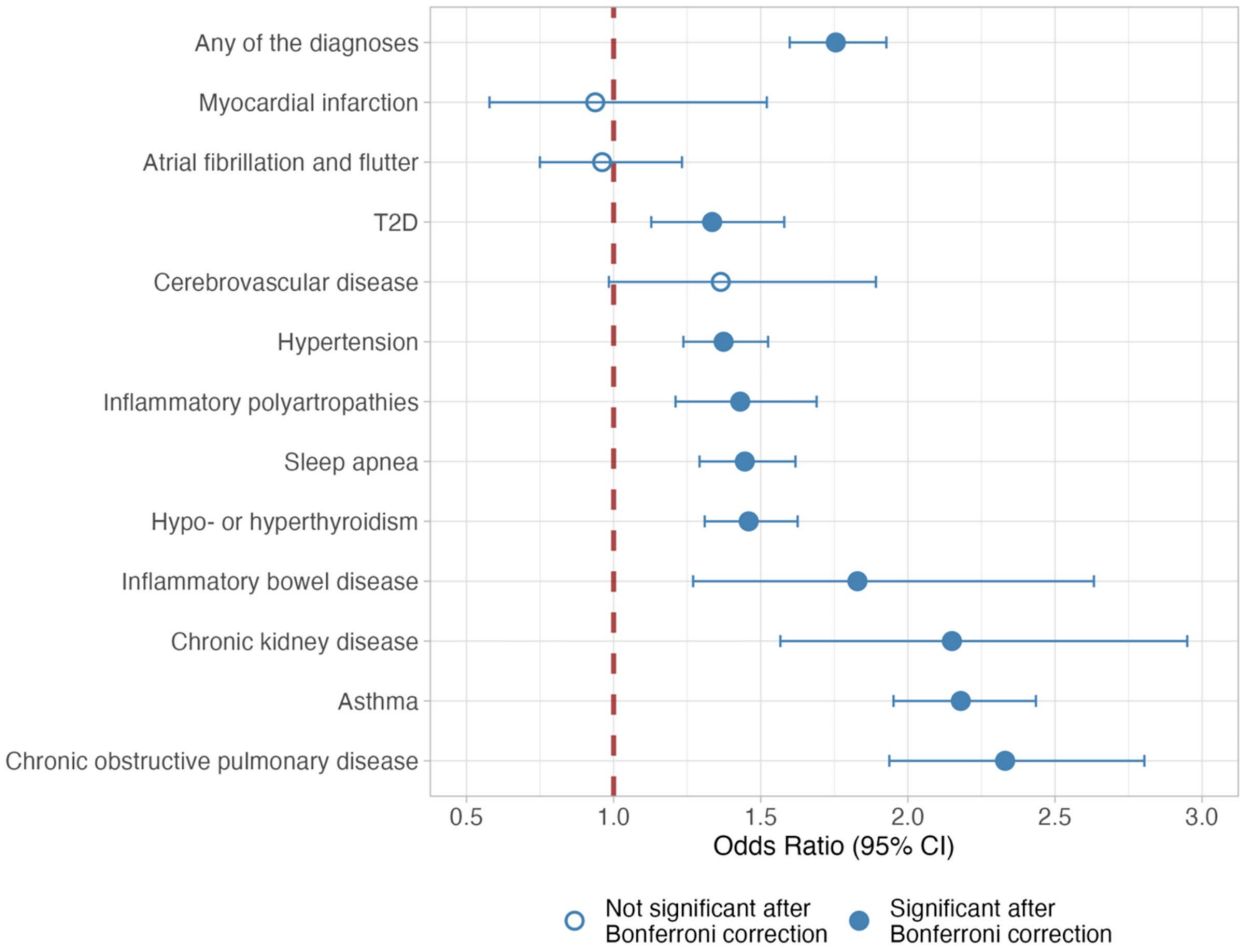
Association of pre-pandemic NCDs (2010–2019) with subsequent long COVID diagnosis (2020–2023). Odds ratios (95% confidence intervals) from logistic regression models estimating the association between NCD diagnoses recorded between January 1, 2010 and December 31, 2019 and subsequent Long COVID diagnosis between June 2020 and December 2023. The dashed vertical line indicates the null value (OR = 1).

We first assessed whether pre-existing non-communicable diseases (NCDs) diagnosed between 2010 and 2019 were associated with subsequent Long COVID diagnosis during 2020–2023. Individuals with any of the examined NCDs had higher odds of Long COVID (OR = 1.76, 95% CI 1.60–1.93, *p* < 0.0001). Several individual conditions were significantly associated with Long COVID (Figure 1; Supplementary Tables S3). The strongest associations were observed for chronic obstructive pulmonary disease (COPD) (OR = 2.33, 95% CI 1.94–2.80, *p* < 0.0001), asthma (OR = 2.18, 95% CI 1.95–2.44, *p* < 0.0001), and chronic kidney disease (OR = 2.15, 95% CI 1.57–2.95, *p* < 0.0001). Other significant associations included sleep apnea (OR = 1.45, 95% CI 1.29–1.61, *p* < 0.0001), thyroid disease (OR = 1.45, 95% CI 1.31–1.63, *p* < 0.0001), inflammatory polyarthropathies (OR = 1.43, 95% CI 1.21–1.69, *p* < 0.0001), hypertension (OR = 1.37, 95% CI 1.24–1.53, *p* < 0.0001), and T2D (OR = 1.33, 95% CI 1.13–1.58, *p* = 0.0008). When additionally adjusting for BMI, the overall pattern of associations remained largely unchanged, except for T2D (OR = 1.20, 95% CI 1.01–1.43, *p* = 0.0391), which no longer met the Bonferroni-corrected threshold (Supplementary Figure S1; Supplementary Table S4).

### Association between Long COVID and new-onset NCDs diagnosed after 2020

**Figure 2:**
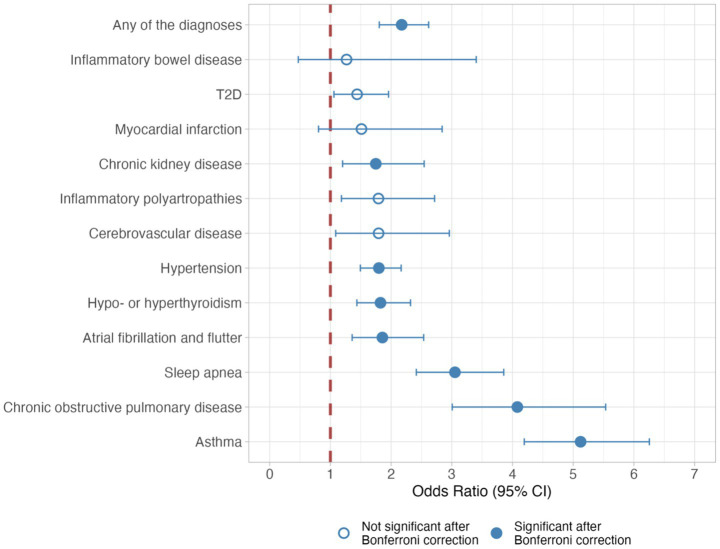
Association between Long COVID diagnosis and first-recorded NCDs during 2021–2023. Odds ratios (95% confidence intervals) for first-recorded NCD diagnoses between January 1, 2021 and December 31, 2023 among individuals with Long COVID compared to participants without a Long COVID diagnosis. The dashed vertical line represents the null value (OR = 1).

We next examined whether individuals with Long COVID had increased odds of receiving first-recorded NCD diagnoses during the pandemic period (2021–2023). Long COVID diagnosis was associated with higher odds of several NCDs compared with individuals without Long COVID (Figure 2; Supplementary Tables S5). The strongest associations were observed for asthma (OR = 5.12, 95% CI 4.19–6.25, *p* < 0.0001) and COPD (OR = 4.08, 95% CI 3.01–5.53, *p* < 0.0001). Elevated odds were also observed for sleep apnea (OR = 3.05, 2.46–3.86, *p* < 0.0001), atrial fibrillation (OR = 1.86, 95% CI 1.36–2.54, *p* = 0.0001), thyroid diseases (OR = 1.83, 95% CI 1.44–2.32, *p* < 0.0001) and hypertension (OR = 1.80, 95% CI 1.49–2.17, *p* < 0.0001), and any of the diseases combined (OR = 2.17, 95% CI 1.80–2.62, *p* < 0.0001). The association with T2D was nominally significant but did not survive Bonferroni correction (OR = 1.44, 95% CI 1.06–1.96, *p* = 0.0201). In the BMI-adjusted model, results were unchanged overall, except for inflammatory polyarthropathies, which reached Bonferroni-corrected significance (Supplementary Figure S2; Supplementary Tables S6). Higher age, female sex, and being overweight or obese were also associated with increased odds of Long COVID (Supplementary Figure S3).

### Increase in new-onset T2D diagnoses following the COVID-19 pandemic

**Figure 3:**
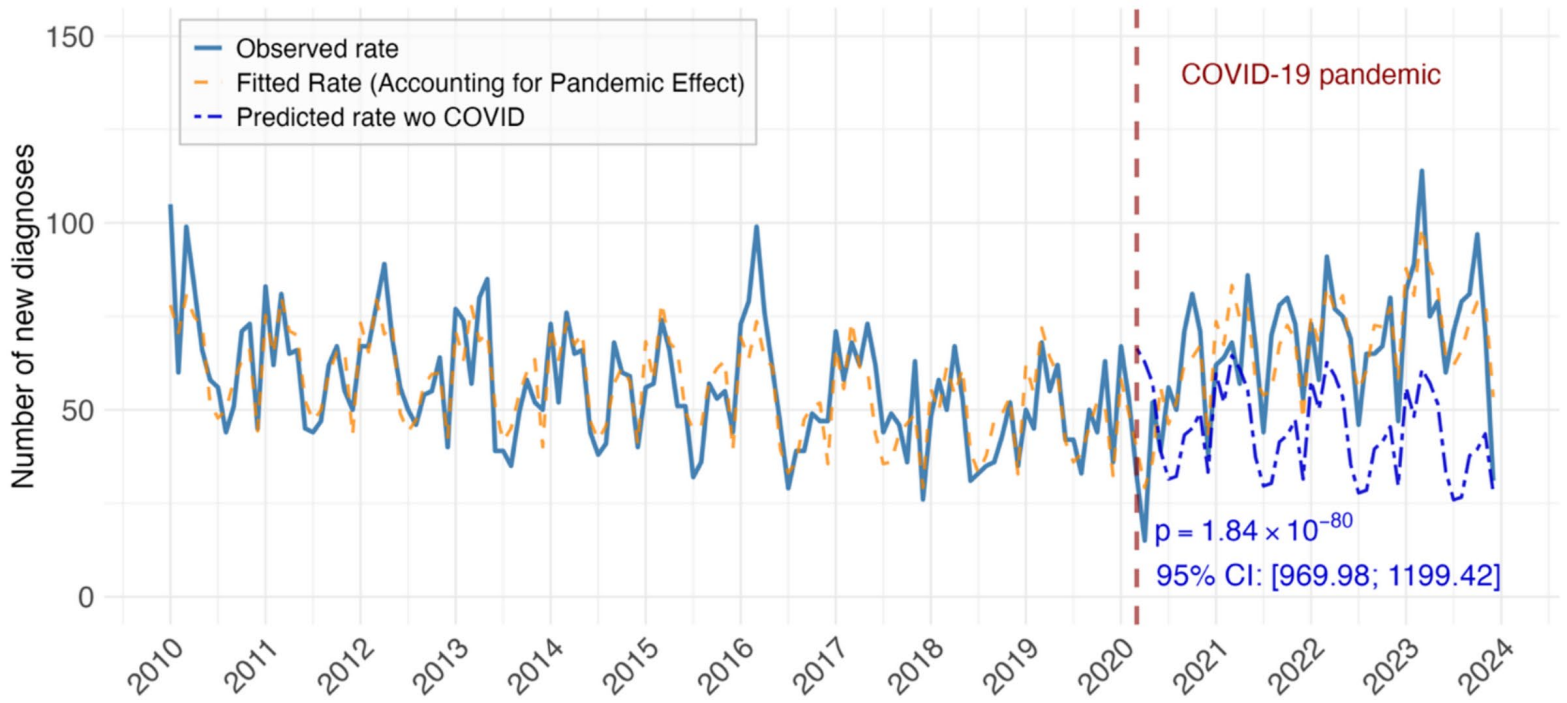
Interrupted time-series analysis of monthly incident T2D diagnoses before and during the COVID-19 pandemic (2012–2023). Observed and model-predicted monthly counts of newly diagnosed T2D. The dashed red vertical line indicates March 2020, marking the beginning of the COVID-19 pandemic period in Estonia. The solid blue line represents observed monthly diagnoses. The orange line represents the SARIMA model fitted to the observed data including pandemic effects. The dashed blue line represents the counterfactual prediction of T2D diagnoses in the absence of the pandemic. The *p* value corresponds to a test of whether the cumulative contribution of the COVID 19 intervention terms (pulse, step, and ramp) from March 2020 to December 2023 differs from zero, whereas the 95% confidence interval quantifies the plausible range for the total cumulative increase in incident T2D diagnoses attributable to these effects over the same period.

To evaluate population-level changes in T2D incidence, we performed an interrupted time-series analysis using monthly counts of newly diagnosed T2D cases from 2010 to 2023. The SARIMA model was fitted to pre-pandemic data (January 2012–February 2020) to estimate the expected counterfactual trajectory. Observed T2D diagnoses during the pandemic period exceeded model-based projections, indicating a significant increase in incidence beyond expected temporal and seasonal trends (*p* = 1.84 × 10^−80^) (Figure 3; Supplementary Figure S4). These results suggest an excess burden of newly diagnosed T2D during the COVID-19 pandemic period.

### Association between COVID-19 polygenic risk score and incident T2D

**Figure 4:**
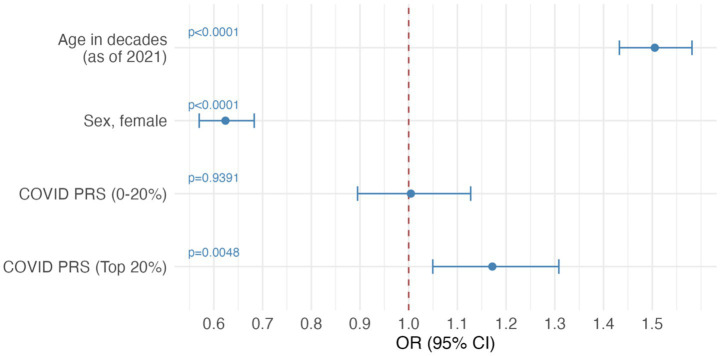
Association between COVID-19 polygenic risk score and incident T2D. Odds ratios (95% confidence intervals) for incident T2D according to COVID-19 PRS quantiles. Models were adjusted for birth year and sex. The dashed vertical line represents the null value (OR = 1). The intermediate PRS group (20–80%) was used as the reference category.

Finally, we assessed whether genetic susceptibility to severe COVID-19 was associated with incident T2D during the pandemic period. Participants were stratified by polygenic risk score (PRS) for severe COVID-19. Individuals in the highest PRS quantile (top 20%) had higher odds of incident T2D than those in the middle PRS group (20–80%), when adjusted for birth year and sex (OR = 1.17, 95% CI 1.05–1.31, *p* = 0.0048) (Figure 4). The association remained significant in the sensitivity analysis that additionally included BMI as a covariate (OR = 1.13, 95% CI 1.01–1.26, *p* = 0.0385) (Supplementary Figure S5). These results suggest that genetic predisposition to severe COVID-19 is linked with incident T2D.

## Discussion

In this population-based study of more than 210,000 EstBB participants with linked electronic health records, we examined clinical and epidemiological associations between Long COVID and NCDs. We observed three key findings. First, several pre-existing NCDs, such as chronic respiratory diseases, chronic kidney disease, hypertension, and T2D, were associated with increased odds of Long COVID diagnosis. Second, individuals with Long COVID had higher odds of receiving first-recorded diagnoses of several NCDs during the pandemic period. Third, population-level incidence of newly diagnosed T2D exceeded expected trends during the COVID-19 pandemic, and genetic susceptibility to severe COVID-19 was associated with increased risk of incident T2D.

Our findings are consistent with previous reports showing that individuals with pre-existing cardiometabolic and respiratory diseases have an increased risk of Long COVID. Conditions such as COPD, asthma, chronic kidney disease, and hypertension may predispose individuals to persistent post-infectious symptoms through mechanisms including systemic inflammation, immune dysregulation, and endothelial dysfunction (3, 29–31). We also observed increased odds of several NCD diagnoses among individuals with Long COVID during the pandemic period, with the strongest associations for respiratory diseases such as asthma and COPD. These patterns are consistent with evidence that COVID-19 may contribute to persistent respiratory and cardiometabolic complications. Although pre-existing T2D was associated with increased odds of Long COVID, the association between Long COVID and incident T2D did not remain significant after multiple testing correction. This discrepancy may reflect limited statistical power for incident T2D cases, a short follow-up period for incident T2D, changes in healthcare accessibility, or that the development of T2D following COVID-19 may be more strongly influenced by broader population-level or pandemic-related factors rather than Long COVID itself. Also, the observed associations may partly reflect increased healthcare use or diagnostic surveillance among individuals with ongoing symptoms.

Our interrupted time-series analysis demonstrated a significant increase in newly diagnosed T2D after the onset of the COVID-19 pandemic compared with expected trends based on pre-pandemic data. Similar increases in T2D incidence following COVID-19 have been reported in large population-based studies and meta-analyses (7–10, 13). Several mechanisms may contribute to this phenomenon, including direct viral effects on pancreatic *β*-cells, systemic inflammation, and metabolic disturbances following infection. However, broader pandemic-related factors may also have played a role, including lifestyle changes, reduced physical activity, psychological stress, weight gain, and altered access to healthcare services (32).

We also found that the higher PRS for severe COVID-19 is associated with an increased likelihood of receiving a first diagnosis of T2D during the post-pandemic period. Previous research has shown the potential of T2D PRS to estimate COVID-19 severity and mortality (33). Our study extends this knowledge by demonstrating that individuals with higher genetic risk for severe COVID-19 are also more likely to develop T2D post-infection. However, this association should be interpreted with caution, as it may reflect horizontal pleiotropy rather than a causal relationship between severe COVID-19 susceptibility and T2D risk.

This study has several strengths. The EstBB provides a large population-based cohort with comprehensive linkage to national healthcare registries, enabling long-term follow-up and reliable ascertainment of clinical diagnoses. The integration of epidemiological and genetic data allowed us to explore potential biological links between COVID-19 severity and metabolic disease risk. In addition, the interrupted time-series design enabled evaluation of population-level changes in T2D incidence while accounting for underlying temporal and seasonal trends.

Several limitations should be considered. Long COVID was identified using ICD-10 codes recorded in routine clinical practice, and diagnostic practices may have varied during the pandemic. Associations between Long COVID and subsequent NCD diagnoses may partly reflect increased healthcare utilization or surveillance bias. The exclusion of 2020 as a washout period to reduce potential misclassification may have led to underestimation of short-term incident NCD cases occurring early in the pandemic. The interrupted time-series analysis captures population-level changes during the pandemic but cannot isolate the causal effects of SARS-CoV-2 infection. Finally, although the PRS findings suggest shared genetic susceptibility, pleiotropy and residual confounding cannot be excluded.

## Data Availability

The data analyzed in this study is subject to the following licenses/restrictions: Genetic predisposition to severe COVID-19 was quantified using a polygenic risk score (PRS) derived from GWAS summary statistics for the B2 phenotype from the COVID-19 Host Genetics Initiative. Summary statistics from the meta-analysis were used. All EstBB participants were genotyped using Illumina GSAv1.0 and GSAv2.0 arrays, as well as GSAv2.0_EST and GSAv3.0_EST arrays with add-on SNV content from EstBB whole-genome sequencing data, aligned to the GRCh37 reference genome. Individual-level data at EstBB can only be accessed through EstBB. Requests to access these datasets should be directed to https://genomics.ut.ee/en/content/Estonian-biobank.

## Group member of Estonian Biobank Research Team

Andres Metspalu, Lili Milani, Tõnu Esko, Reedik Mägi, Mait Metspalu, Mari Nelis and Georgi Hudjashov.
