# Supplementary material for: Bidirectional associations between chronic diseases and Long COVID: a population-based cohort and interrupted time-series analysis
Source: Front Public Health. 2026 Jul 16;14:1834448. doi: 10.3389/fpubh.2026.1834448 (PMC13422490; doi:10.3389/fpubh.2026.1834448)
Supplement: Supplementary file 1 [file Supplementary_File_1.pdf]

Supplemental Material

Supplementary Table 1. Age and BMI distributions of individuals with and without Long COVID.

	**Long COVID**	**Controls**
Male	652	67 874
Female	1987	131 297
Age Mean (SD)	49.5 (15.7)	46.4 (15.9)
Age Median (IQR)	49 (38-61)	45 (33-58)
BMI Mean (SD)	27.5 (6.0)	26.5 (5.5)

Supplementary Table 2. Age groups of individuals with Long COVID.

**Age Group (Years)**	**Male (%)**	**Female (%)**	**Total (%)**
17-29	46 (1.7)	144 (5.5)	190 (7.2)
30-39	97 (3.7)	335 (12.7)	432 (16.4)
40-49	124 (4.7)	445 (16.9)	569 (21.6)
50-59	151 (5.7)	406 (15.4)	557 (21.1)
60-69	133 (5.0)	351 (13.3)	484 (18.3)
70-79	66 (2.5)	211 (8.0)	277 (10.5)
80+	25 (1.3)	95 (3.6)	130 (4.9)
**Total (%)**	**652 (24.7)**	**1987 (75.3)**	**2639 (100)**

Supplementary Table 3. Number of individuals with each diagnosis during 2010–2019 and the number of long COVID diagnoses among them in 2020–2023. Adjusted odds ratios (ORs) were obtained from logistic regression models adjusting for birth year and sex. The composite row “Any of these diagnoses” includes all ICD10 codes listed above. Because 12 diagnostic groups were evaluated, multiple testing was accounted for using the Bonferroni correction, yielding a significance threshold of α = 0.0042 (0.05/12). p-values below this threshold were considered statistically significant.

**Diagnosis**	**ICD10 codes**	**Individuals with diagnosis in 2010-2019**	**Long COVID among them**	**OR**	**P-value**
Type 2 diabetes	E11	9218	160	1.33	0.0008
Hypertension and related disorders	I10, I11, I12, I13, I15	49702	758	1.37	<0.0001
Mycocardial infarction	I21, I22	1501	17	0.94	0.7930
Cerebrovascular disease	I63, I64, I69	2108	38	1.36	0.0626
Artial fibrillation and flutter	I48	5285	68	0.96	0.7530
Inflammatory polyarthropathies	M05, M06, M07, M08, M09	8332	154	1.43	<0.0001
Sleep apnea	G47.3	23150	394	1.45	<0.0001
Thyroid diseases	E03, E04, E05	25037	457	1.45	<0.0001
Inflammatory bowel disease	K50, K51	1440	30	1.83	0.0011
Chronic kidney disease	N18, N19	1507	41	2.15	<0.0001
Asthma	J45	16499	392	2.18	<0.0001
Chronic obstructive and related pulmonary diseases	J43, J44	4547	129	2.33	<0.0001
Any of these diagnoses	All codes above	88550	1367	1.76	<0.0001

Supplementary Table 4. Number of individuals with each diagnosis during 2010–2019 and the number of long COVID diagnoses among them in 2020–2023. Adjusted odds ratios (ORs) were obtained from logistic regression models adjusting for age birth year, sex, and BMI. The composite row “Any of these diagnoses” includes all ICD10 codes listed above. Because 12 diagnostic groups were evaluated, multiple testing was accounted for using the Bonferroni correction, yielding a significance threshold of α = 0.0042 (0.05/12). p values below this threshold were considered statistically significant. The number of cases included in this analysis is smaller than in the main analysis because BMI measurements were not available for all individuals.

**Diagnosis**	**ICD10 codes**	**Individuals with diagnosis in 2010-2019**	**Long COVID among them**	**OR**	**P-value**
Type 2 diabetes	E11	8988	156	1.20	0.0391
Hypertension and related disorders	I10, I11, I12, I13, I15	48407	745	1.32	<0.0001
Mycocardial infarction	I21, I22	1464	17	0.94	0.8062
Cerebrovascular disease	I63, I64, I69	2057	38	1.37	0.0567
Artial fibrillation and flutter	I48	5125	67	0.94	0.6177
Inflammatory polyarthropathies	M05, M06, M07, M08, M09	8089	151	1.44	<0.0001
Sleep apnea	G47.3	22437	379	1.41	<0.0001
Thyroid diseases	E03, E04, E05	24378	442	1.44	<0.0001
Inflammatory bowel disease	K50, K51	1404	29	1.85	0.0012
Chronic kidney disease	N18, N19	1480	41	2.11	<0.0001
Asthma	J45	15956	380	2.15	<0.0001
Chronic obstructive and related pulmonary diseases	J43, J44	4435	129	2.35	<0.0001
Any of these diagnoses	All codes above	85970	1324	1.73	<0.0001

Supplementary Table 5. Number of individuals with Long COVID and the number of first-time diagnoses of each condition in 2021–2023. Odds ratios (ORs) and p-values are obtained from logistic regression models adjusted for birth year and sex. Individuals with any record of the respective diagnosis prior to 2021 were excluded from the analysis. The composite outcome "Any of these diagnoses" includes all ICD-10 codes listed above. Because 12 diagnostic groups were evaluated, multiple testing was accounted for using the Bonferroni correction, yielding a significance threshold of $\alpha = 0.0042$ (0.05/12). p-values below this threshold were considered statistically significant.

Diagnosis	ICD10 codes	Individuals with Long COVID	First-time diagnosis among them	OR	P-value
Type 2 diabetes	E11	2071	43	1.44	0.0201
Hypertension and related disorders	I10, I11, I12, I13, I15	1406	135	1.80	<0.0001
Mycocardial infarction	I21, I22	2225	10	1.51	0.1990
Cerebrovascular disease	I63, I64, I69	2200	16	1.79	0.0220
Artial fibrillation and flutter	I48	2158	43	1.86	0.0001
Inflammatory polyarthropathies	M05, M06, M07, M08, M09	2047	23	1.79	0.0060
Sleep apnea	G47.3	2103	76	3.05	<0.0001
Thyroid diseases	E03, E04, E05	1684	72	1.83	<0.0001
Inflammatory bowel disease	K50, K51	2210	4	1.27	0.6397
Chronic kidney disease	N18, N19	2204	29	1.75	0.0035
Asthma	J45	1831	108	5.12	<0.0001
Chronic obstructive and related pulmonary diseases	J43, J44	2082	45	4.08	<0.0001
Any of these diagnoses	All codes above	885	143	2.17	<0.0001

Supplementary Table 6. Number of individuals with Long COVID and the number of first-time diagnoses of each condition in 2021–2023. Odds ratios (ORs) and p-values are obtained from logistic regression models adjusted for birth year, sex, and BMI. Individuals with any record of the respective diagnosis prior to 2021 were excluded from the analysis. The composite outcome “Any of these diagnoses” includes all ICD-10 codes listed above. Because 12 diagnostic groups were evaluated, multiple testing was accounted for using the Bonferroni correction, yielding a significance threshold of α = 0.0042 (0.05/12). p-values below this threshold were considered statistically significant. The number of cases included in this analysis is smaller than in the main analysis because BMI measurements were not available for all individuals.

**Diagnosis**	**ICD10 codes**	**Individuals with Long COVID**	**First-time diagnosis among them**	**OR**	**P-value**
Type 2 diabetes	E11	1983	43	1.39	0.0390
Hypertension and related disorders	I10, I11, I12, I13, I15	1333	125	1.68	<0.0001
Mycocardial infarction	I21, I22	2133	10	1.54	0.1803
Cerebrovascular disease	I63, I64, I69	2108	16	1.83	0.0179
Artial fibrillation and flutter	I48	2068	42	1.82	0.0002
Inflammatory polyarthropathies	M05, M06, M07, M08, M09	1963	23	1.84	0.0040
Sleep apnea	G47.3	2012	71	2.87	<0.0001
Thyroid diseases	E03, E04, E05	1610	68	1.81	<0.0001
Inflammatory bowel disease	K50, K51	2119	4	1.30	0.6028
Chronic kidney disease	N18, N19	2112	29	1.76	0.0031
Asthma	J45	1749	106	5.19	<0.0001
Chronic obstructive and related pulmonary diseases	J43, J44	1991	43	3.99	<0.0001
Any of these diagnoses	All codes above	838	135	2.12	<0.0001

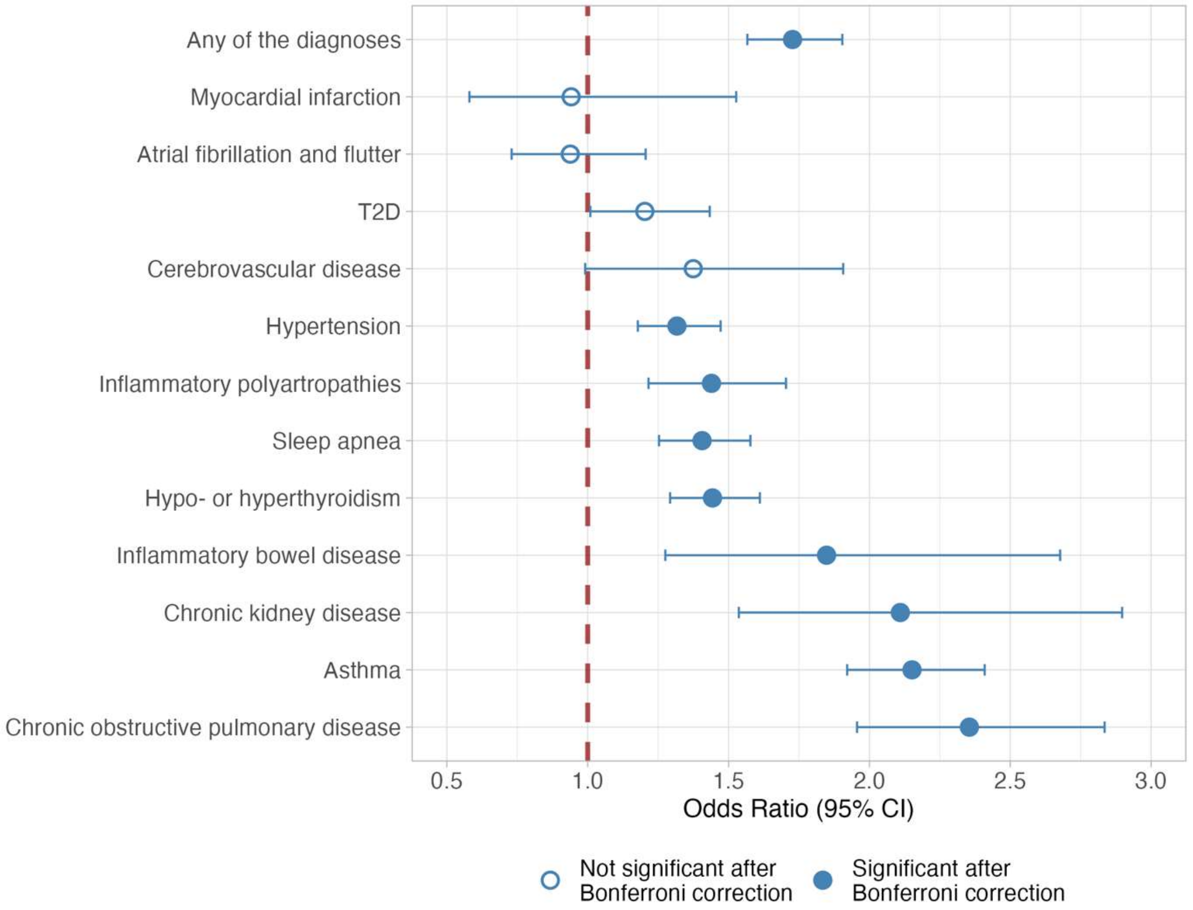

Supplementary Figure 1. Association of pre pandemic NCDs (2010–2019) with subsequent Long COVID diagnosis (2020–2023), adjusted for birth year, sex, and BMI. Odds ratios (and 95% confidence intervals) from logistic regression models estimating the association between NCD diagnoses recorded between January 1, 2010 and December 31, 2019 and subsequent Long COVID diagnosis between June 2020 and December 2023. The dashed vertical line indicates the null value (OR = 1). Filled points indicate associations significant after Bonferroni correction ($p < 0.05/12$); hollow points indicate non significant associations.

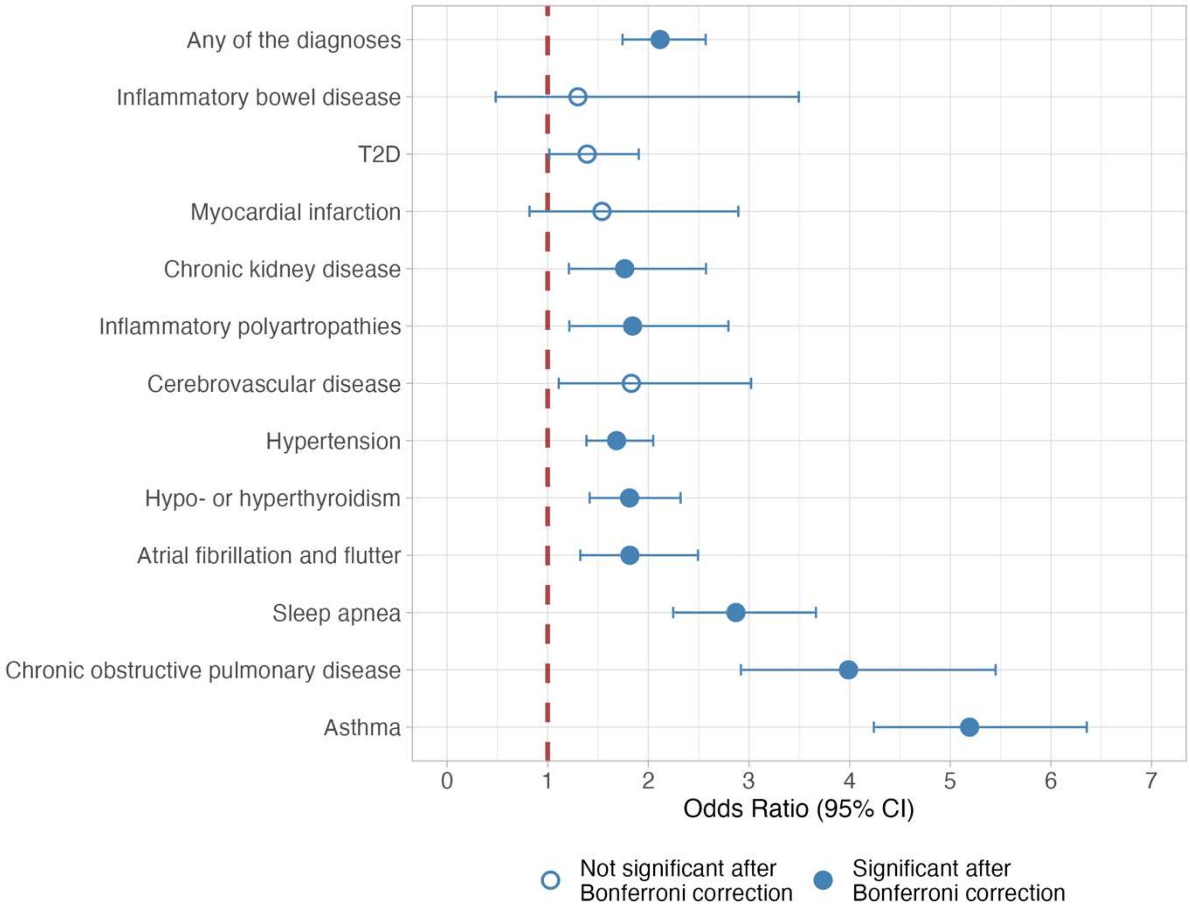

Supplementary Figure 2. Association between Long COVID diagnosis and first-recorded NCDs during 2021–2023, adjusted for birth year, sex, and BMI. Odds ratios (and 95% confidence intervals) from logistic regression models estimating the association between NCD diagnoses recorded between January 1, 2010 and December 31, 2019 and subsequent Long COVID diagnosis between June 2020 and December 2023. The dashed vertical line indicates the null value (OR = 1). Filled points indicate associations significant after Bonferroni correction ($p < 0.05/12$); hollow points indicate non significant associations.

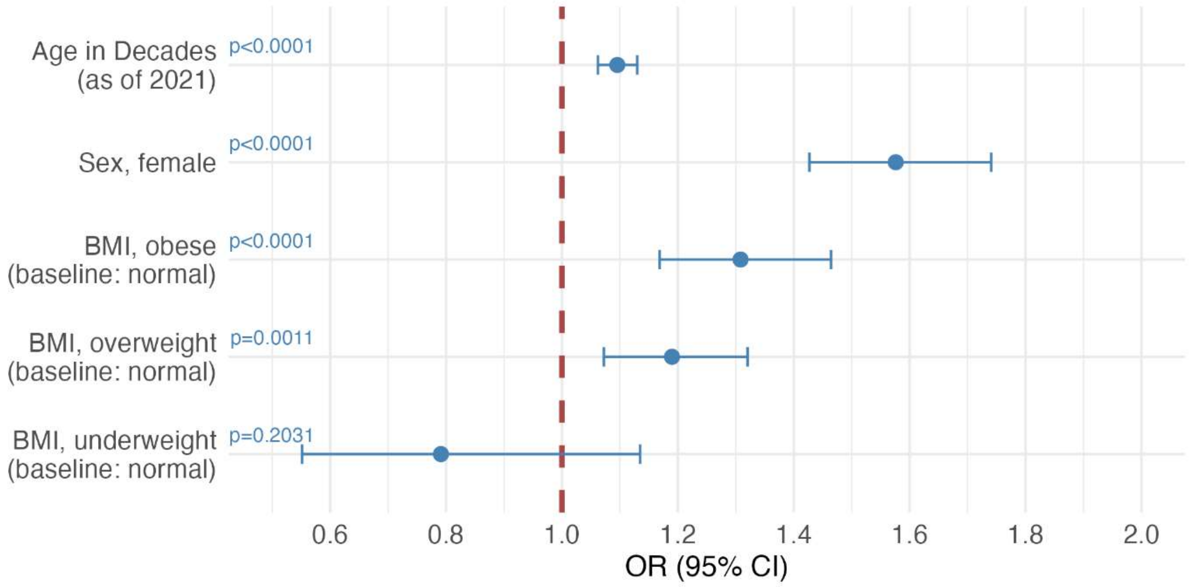

Supplementary Figure 3. Association between BMI and Long COVID diagnosis. Association between BMI and odds of Long COVID diagnosis among EstBB participants. Estimates are derived from regression models adjusted for birth year and sex. Points represent odds ratios and lines represent 95% confidence intervals.

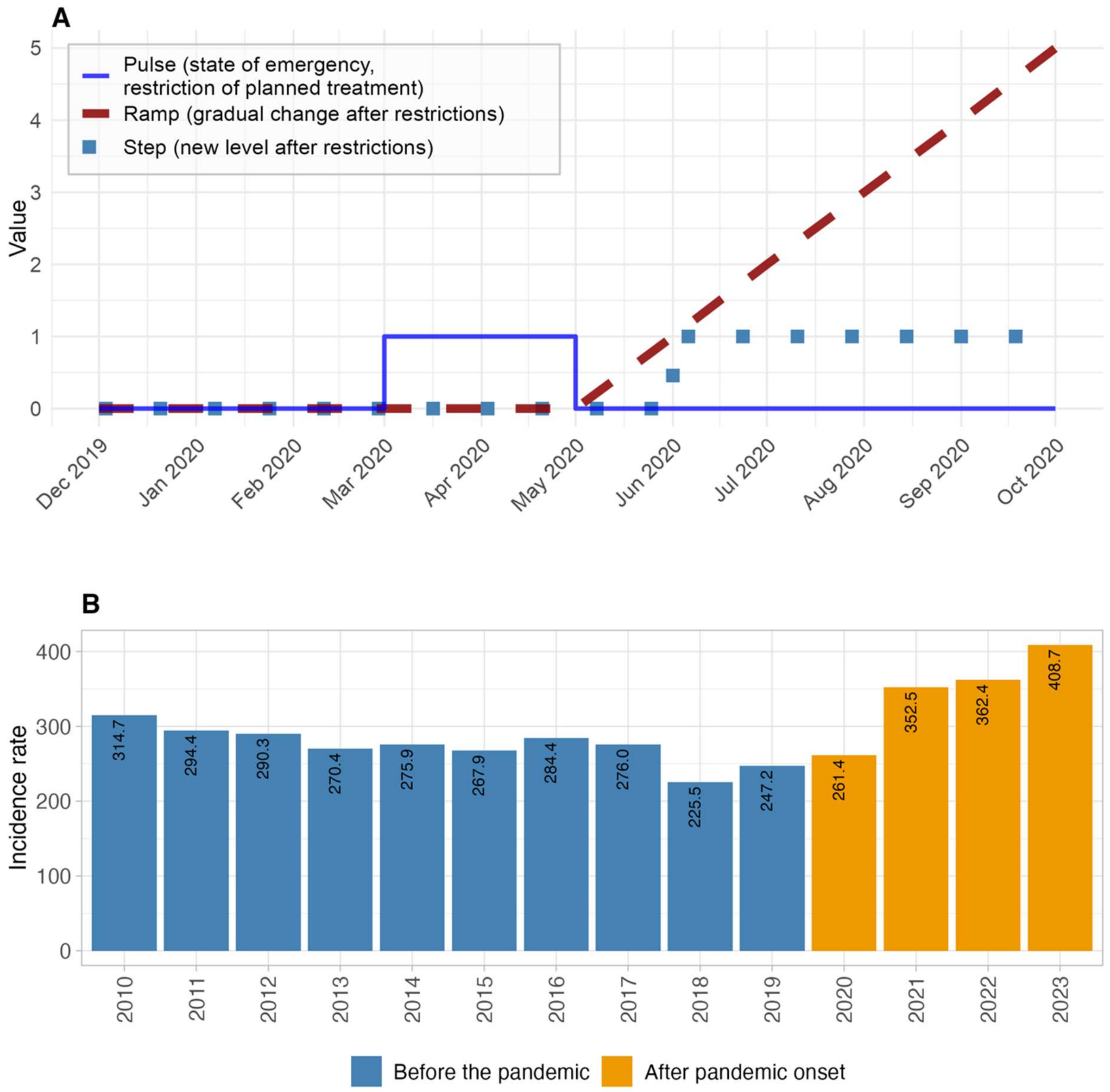

Supplementary Figure 4. A) Illustration of the modeled intervention effects used in the interrupted time-series analysis. The pulse term represents the short-term disruption at the onset of the COVID-19 pandemic, the step term reflects an immediate and sustained shift in the incidence level, and the ramp term captures a gradual change in the underlying trend following the pandemic onset. **(B)** Annual incidence rate of newly diagnosed type 2 diabetes (T2D) among living biobank participants aged 45–80 years from 2010 to 2023. Bars are stratified into pre-pandemic (2010–2019) and post-pandemic onset (2020–2023) periods. Incidence rates were calculated per 100,000 individuals at risk each year.

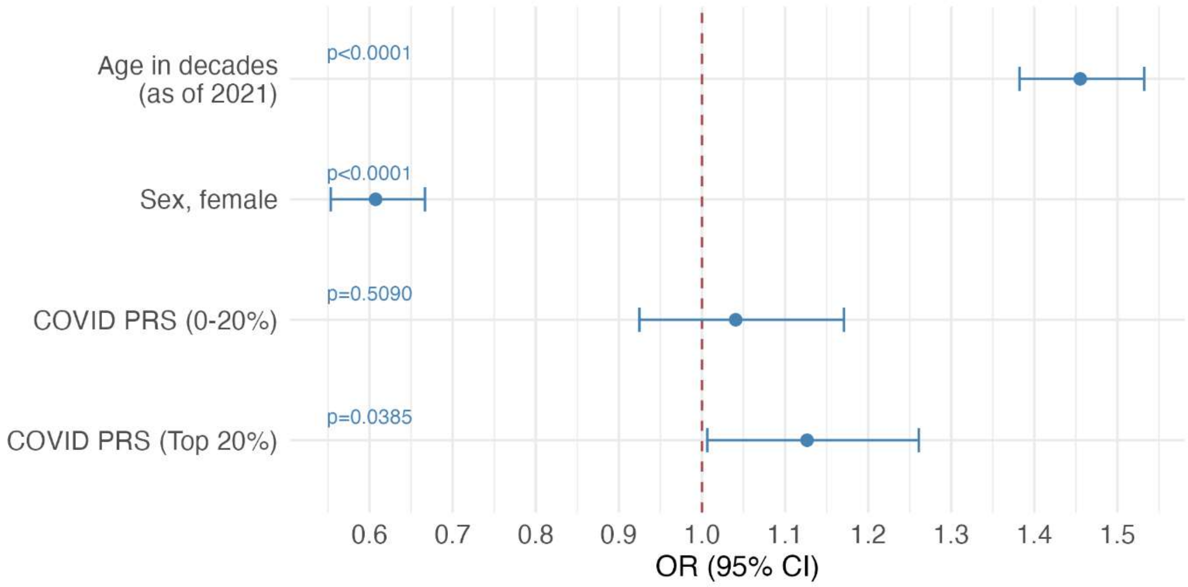

Supplementary Figure 5. Association between COVID-19 polygenic risk score and incident T2D, adjusted for birth year, sex, first 4 genotype principal components, and BMI. The first 4 genotype principal compon
